# Treosulfan-induced myalgia in pediatric hematopoietic stem cell transplantation identified by an electronic health record text mining tool

**DOI:** 10.1038/s41598-021-98669-3

**Published:** 2021-09-27

**Authors:** M. Y. Eileen C. van der Stoep, Dagmar Berghuis, Robbert G. M. Bredius, Emilie P. Buddingh, Alexander B. Mohseny, Frans J. W. Smiers, Henk-Jan Guchelaar, Arjan C. Lankester, Juliette Zwaveling

**Affiliations:** 1 Department of Clinical Pharmacy and Toxicology, Leiden University Medical Center, PO Box 9600, 2300 RC Leiden, Leiden, The Netherlands; 2 Department of Pediatrics, Leiden University Medical Center, Leiden, The Netherlands

**Keywords:** Pain, Paediatric research, Adverse effects, Chemotherapy

## Abstract

Treosulfan is increasingly used as myeloablative agent in conditioning regimen prior to allogeneic hematopoietic stem cell transplantation (HSCT). In our pediatric HSCT program, myalgia was regularly observed after treosulfan-based conditioning, which is a relatively unknown side effect. Using a natural language processing and text-mining tool (CDC), we investigated whether treosulfan compared with busulfan was associated with an increased risk of myalgia. Furthermore, among treosulfan users, we studied the characteristics of given treatment of myalgia, and studied prognostic factors for developing myalgia during treosulfan use. Electronic Health Records (EHRs) until 28 days after HSCT were screened using the CDC for myalgia and 22 synonyms. Time to myalgia, location of pain, duration, severity and drug treatment were collected. Pain severity was classified according to the WHO pain relief ladder. Logistic regression was performed to assess prognostic factors. 114 patients received treosulfan and 92 busulfan. Myalgia was reported in 37 patients; 34 patients in the treosulfan group and 3 patients in the busulfan group (p = 0.01). In the treosulfan group, median time to myalgia was 7 days (0–12) and median duration of pain was 19 days (4–73). 44% of patients needed strong acting opiates and adjuvant medicines (e.g. ketamine). Hemoglobinopathy was a significant risk factor, as compared to other underlying diseases (OR 7.16 95% CI 2.09–30.03, p = 0.003). Myalgia appears to be a common adverse effect of treosulfan in pediatric HSCT, especially in hemoglobinopathy. Using the CDC, EHRs were easily screened to detect this previously unknown side effect, proving the effectiveness of the tool. Recognition of treosulfan-induced myalgia is important for adequate pain management strategies and thereby for improving the quality of hospital stay.

## Introduction

Treosulfan, a bifunctional alkylating agent, was originally registered for the palliative treatment of ovarian carcinoma in the mid-90 s (Ovastat^®^)^1–4^. More recently in 2019, it was also registered as part of conditioning treatment prior to allogeneic hematopoietic stem cell transplantation (alloHSCT) in adult and pediatric patients (Trecondi^®^)^5^. In the past decade, several studies have reported the efficacy and tolerability of treosulfan-based conditioning regimens in pediatric alloHSCT for both non-malignant and malignant diseases^6–12^. Treosulfan-based conditioning has gained popularity, particularly in children with non-malignant diseases, because of its favorable toxicity profile. Common side effects are gastrointestinal, mucosal and skin disorders and elevation of liver enzymes, but they are usually limited and mild. In our pediatric HSCT program, some patients experienced myalgia and arthralgia after conditioning with treosulfan, side effects which are not mentioned in the Summary of Product Characteristics (SmPC) of Ovastat^®^^13^. In the SmPC of Trecondi^®^, that has become recently available, pain in extremities is mentioned in the undesirable effects in the pediatric population with unknown frequency^14^. In the European pharmacovigilance database (EudraVigilance, www.adrreports.eu), there are nine reports within the group ‘musculoskeletal and connective tissue disorders’, of 304 reports up to the end of 2020. The majority of these reports are in adult patients.

Real world data might contribute to the knowledge on adverse events and the electronic health record (EHR) is an important source of data and contains valuable information collected during routine clinical practice, including side effects of drugs that the patient experiences during treatment. Unfortunately, this information is often stored in the EHR as free-text notes and therefore less suitable for automated extraction. Manual chart review is the gold standard for collection of data from EHRs, but this is laborious and very time-consuming^15^. Natural language processing (NLP) and text mining techniques in the EHR can provide additional information about drugs that has not been discovered in clinical development. Recently, the NLP and text-mining tool Clinical Data Collector (CDC; CTcue B.V., Amsterdam, The Netherlands), has proven to be a helpful tool for retrieving real world data (RWD) from EHRs in a validation study in patients with metastatic renal cell carcinoma, compared to manual chart review^16^. With the use of CDC, we investigated whether treosulfan compared with busulfan was associated with an increased risk of myalgia. Furthermore, among treosulfan users, we studied the characteristics of given treatment of myalgia, and studied prognostic factors for developing myalgia during treosulfan use.

## Methods

### Study population and design

A retrospective cohort study was conducted at the Pediatric Hematopoietic Stem Cell Transplantation unit of the Leiden University Medical Centre (LUMC) from May 2011 until May 2019. Pediatric patients (≤ 18 years) who received treosulfan (TREO)- or busulfan (BU)-based conditioning prior to HSCT were included and divided in two cohorts. Treosulfan was given in a dose of 42 g/m^2^ and 30 g/m^2^ in children above or under the age of 1 year old, respectively. Busulfan was initially dosed as 120 mg/m^2^ and then targeted to a total exposure (as area under the concentration curve, AUC_0-∞_) of 75–95 mg*h/L.

### Data retrieval

Electronic Health Records (EHRs) were screened anonymously, using the intelligent search engine CTcue Clinical Data Collector (CTcue B.V., Amsterdam, The Netherlands), a software package that can be used to search through Electronic Health Records^16^. The EHR includes records from nurses, physicians, physical therapists, dieticians, social workers and pharmacists. The patient population and data points are defined using CDC queries. Two queries were created; in one patients were included ≤ 18 years of age that have received treosulfan and in the other patients received busulfan between May 2011 and May 2019. Using myalgia and 22 synonyms as keywords (see Supplementary Information), patients with one of these keywords mentioned in the EHR until 28 days after HSCT were highlighted. Subsequently, highlighted EHRs were manually checked for validity.

### Measurement of myalgia and pain severity

The presence of myalgia within 28 days after HSCT (i.e. discomfort originating from a muscle or group of muscles) was scored as an event. Time of onset, location and duration of pain were derived manually from the EHR. Duration of pain was derived from the use of pain medication. Additionally, pain severity was categorized according the World Health Organization (WHO) pain relief ladder: paracetamol (PCM) (step 1), PCM and tramadol (step 2), addition of strong acting opiate (step 3) and addition of adjuvant medicines (e.g. ketamine, clonidine, pregabalin) (step 4). When mucositis was present, EHRs were thoroughly screened to confirm that pain medication was given for myalgia and not for mucositis only.

### Collection of other variables

Patient characteristics such as gender, age at SCT, underlying disease and transplant characteristics such as donor, graft, match and graft versus host disease (GvHD) prophylaxis were collected from the EHRs. Creatine kinase (CK) levels and treosulfan exposure (as AUC_0-∞_) were collected if available.

### Endpoints

The incidence and course (timing, location of pain and duration) of myalgia after conditioning were the primary endpoints. Secondary endpoint was pain severity according to the WHO pain relief ladder. Other variables noted above were collected to evaluate potential predisposing factors.

### Statistical analysis

Descriptive statistics, such as median and frequency, were used to summarize baseline characteristics and outcomes. Odds ratios (OR) were estimated by means of logistic regression to examine the association between conditioning regimen (TREO-based vs. BU-based) and myalgia and adjusted for possible confounding (hemoglobinopathies versus other indications). In the TREO cohort, univariable and multivariable logistic regression was performed to assess whether baseline- or transplant characteristics (age, underlying disease, conditioning regimen, treosulfan exposure) were prognostic for the development of myalgia. All P-values were 2-tailed and considered significant when P < 0.05. Statistical analyses were performed using R version 3.6.1 and RStudio version 1.2.5019.

### Ethics approval

The study was approved by the Medical Ethics Review Committee of the LUMC, Leiden. Informed consent was waived by the Medical Ethics Review Committee of the LUMC. All methods were carried out in accordance with relevant guidelines and regulations.

## Results

### Patients and baseline characteristics

A total of 206 patients were included in the study, 114 patients were treated with treosulfan-based conditioning (TREO cohort) and 92 with busulfan-based conditioning (BU cohort). The median age was 5.4 and 8.5 years old in the TREO and BU cohort, respectively. There were 64 patients under 3 years of age. The majority of patients with hemoglobinopathy (i.e. beta-thalassemia or sickle cell disease (SCD)) received a TREO-based conditioning regimen, whereas patients with a hematological malignancy were mostly treated with a BU-based regimen. The most common combination of conditioning agents within the TREO cohort was treosulfan combined with fludarabine and thiotepa (66.7%), followed by treosulfan with fludarabine alone (31.6%). Within the BU cohort this was busulfan with fludarabine and clofarabine (58.7%), followed by busulfan and fludarabine (30.4%) and busulfan combined with fludarabine and thiotepa (7.6%). Serotherapy and GvHD prophylaxis were comparable among the two groups, except that post-transplant cyclophosphamide (PTCy) was used in a subgroup of the TREO cohort namely when a patient was transplanted with a mismatched donor. Baseline characteristics are summarized in Table 1.

**Table 1:** Patient characteristics.

Characteristic	Treosulfan (n = 114)	Busulfan (n = 92)
Age at SCT (years, median (range))	5.4 (0.2–18.2)	8.5 (0.4–17.8)
Sex (M/F) (%)	62/38	58/42
**Diagnosis for HSCT**		
Beta-thalassemia (%)	35 (30.7)	6 (6.5)
Sickle cell disease (%)	20 (17.5)	0 (0)
Inborn errors of immunity (%)	32 (28.1)	10 (10.9)
Hematological malignancy (%)	18 (15.8)	63 (68.5)
Bone marrow failure (%)	9 (7.9)	13 (14.1)
**Donor**		
MSD (%)	36 (31.6)	18 (19.6)
MUD (≥ 9/10) (%)	62 (54.4)	69 (75.0)
MMFD (haplo) (%)	16 (14.0)	5 (5.4)
**Stem cell source**		
BM (%)	85 (74.6)	63 (68.5)
PBSC (%)	14 (12.3)	14 (15.2)
CB (%)	15 (13.2)	15 (16.3)
**Conditioning**		
Treo-Flu-Thiotepa (%)	76 (66.7)	–
Treo-Flu (%)	36 (31.6)	–
Treo-Other (%)	2 (1.7)	–
Bu-Flu-Clo (%)	–	54 (58.7)
Bu-Flu (%)	–	28 (30.4)
Bu-Flu-Thiotepa (%)		7 (7.6)
Bu-Cy-Mel (%)	–	3 (3.3)
**Serotherapy**		
ATG (%)	77 (67.5)	71 (77.2)
Alemtuzumab (%)	27 (23.7)	7 (7.6)
No (%)	10 (8.8)	14 (15.2)
**GvHD prophylaxis**		
CsA + MTX(%)	60 (52.6)	57 (62.0)
PTCy + MMF + CsA (%)	16 (14.0)	0 (0)
CsA + Pred (%)	9 (7.9)	11 (12.0)
CsA (%)	9 (7.9)	3 (3.3)
Other (%)	13 (11.4)	16 (17.4)
None (%)	7 (6.1)	5 (5.4)

*MSD* matched sibling donor, *MUD* matched unrelated donor, *MMFD* mismatched family donor, *BM* bone marrow, *PBSC* peripheral blood stem cells, *CB* cord blood, *Treo* treosulfan, *Flu* fludarabine, *Thio* thiotepa, *Bu* busulfan, *Clo* clofarabine, *ATG* Anti thymocyte globulin, *GvHD* Graft-versus-Host Disease, *CsA* Cyclosporine A, *MTX* methotrexate, *PTCy* Post-transplant cyclophosphamide, *MMF* mycophenolate mofetil, *Pred* prednisolone.

### Incidence, course and duration of myalgia

**Figure 1:**
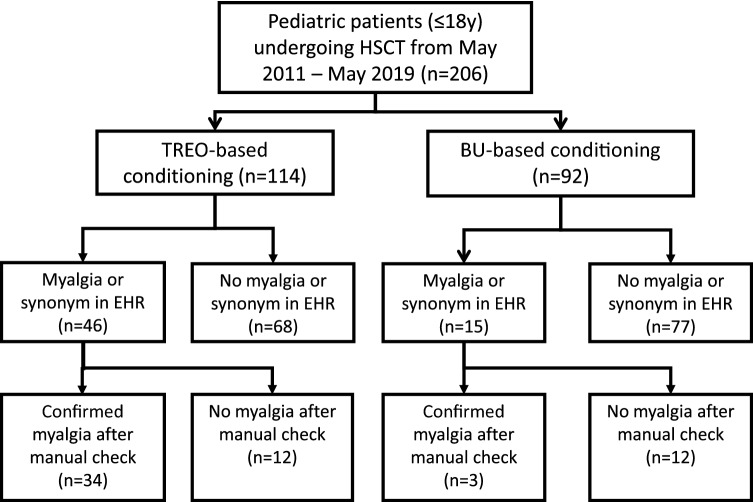
Study design. *HSCT* Hematopoietic stem cell transplantation, *TREO* treosulfan, *BU* busulfan, *EHR* Electronic Health Record.

Myalgia or one of the synonyms were found in 46 of 114 EHRs (40.4%) in the TREO cohort, using the CDC of which 34 patients (29.8%) were confirmed after manual check. In the BU-cohort, 15 out of 92 EHRs (16.3%) were selected using the CDC. Three (3.3%) were confirmed after manual check (Fig. 1). Manual check prevented a denial of an adverse event from being scored as an event. Patients in the TREO cohort were more likely to experience myalgia than patients in the BU cohort (crude OR 12.61, 95% CI 4.32–53.78, p < 0.001; adjusted OR 5.36, 95% CI 1.63–24.19, p = 0.01) . In addition, the 3 patients who experienced myalgia in the BU group, experienced this myalgia during or directly after infusion of clofarabine. Characteristics of patients with myalgia are summarized in Table 2. In the TREO cohort, median time to myalgia was 7 days (range 0–12), calculated from the first day of TREO infusion. The most reported locations of pain were legs (97%) and arms (82%), often combined with pain in knees (47%) and elbows (26%). Other locations in which myalgia was reported were feet (44%), neck (26%), back (24%), hands (24%) and shoulder (21%). In patients under 3 years of age unwillingness to stand, pain and/or crying when stretching or bending arms or legs were considered as a report of pain in legs and arms. Duration of pain varied greatly, ranging from 4 to 73 days, with a median period of 19 days. Almost half of patients (44%) experienced pain for more than 3 weeks. CK-levels were measured in 6 patients during the period of myalgia, which were all within the normal range.

**Table 2:** Myalgia characteristics in the treosulfan cohort and busulfan cohort.

Characteristic	Treosulfan (n = 34)	Busulfan (n = 3)
Age at SCT (years, median (range))	12.2 (1.8–18.2)	11.5 (10.9–17.2)
Sex (n: M/F)	20/14	67/33
**Diagnosis for HSCT**		
Beta-thalassemia (%)	15 (44)	–
Sickle cell disease (%)	13 (38)	–
Inborn errors of immunity (%)	1 (3)	–
Hematological malignancy (%)	4 (12)	3 (100)
Bone marrow failure (%)	1 (3)	–
**Conditioning**		
Treo-Flu-Thiotepa (%)	28 (82)	–
Treo-Flu (%)	5 (15)	–
Treo-Other (%)	1 (3)	–
Bu-Flu-Clo (%)	–	3 (100)
**Location of pain**		
Leg (%)	33 (97)	3 (100)
Arm (%)	28 (82)	3 (100)
Knee / ankle (%)	16 (47)	–
Elbow (%)	9 (26)	–
Neck (%)	9 (26)	–
Back (%)	8 (24)	–
Shoulder (%)	7 (21)	–
Foot (%)	15 (44)	–
Hand (%)	8 (24)	–
**Duration of pain**		
≤ 7 days (%)	4 (12)	1 (33)
8–14 days (%)	7 (20)	2 (67)
15–21 days (%)	8 (24)	–
> 21 days (%)	15 (44)	–
**Medical intervention**		
Paracetamol/acetaminophen	34 (100)	3 (100)
NSAIDs	4 (12)	1 (33)
Tramadol	32 (94)	2 (67)
Opiate, oral	5 (15)	1 (33)
Opiate, intravenous	21 (62)	1 (33)
Antiepileptics (e.g. gabapentin, pregabalin)	8 (24)	–
Other (e.g. ketamine, clonidine, benzodiazepines)	13 (38)	1 (33)
**Pain severity**		
Step 1 (paracetamol)	2 (6)	
Step 2 (paracetamol + tramadol)	11 (32)	1 (33)
Step 3 (addition of strong acting opiate)	6 (18)	1 (33)
Step 4 (addition of adjuvant medicines)	15 (44)	1 (33)

*Treo* treosulfan, *Flu* fludarabine, *Thio* thiotepa, *Bu* busulfan, *Clo* clofarabine.

### Pain severity

**Figure 2:**
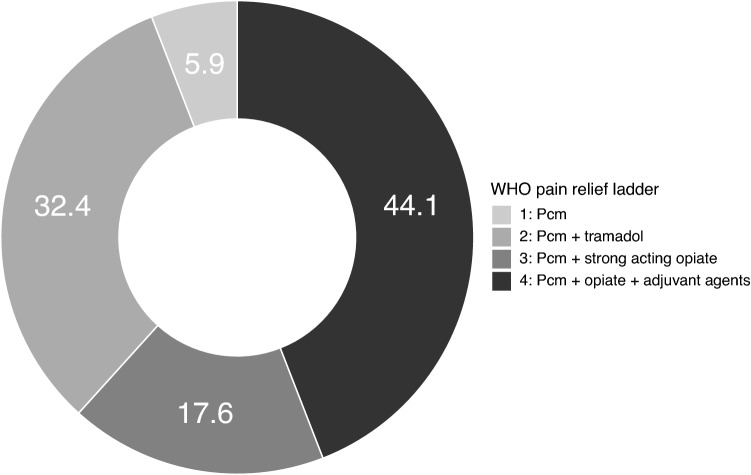
Distribution (in %) of pain severity (classified as steps in the WHO pain relief ladder) in patients with myalgia in the TREO cohort (*Pcm* paracetamol).

Pain severity is shown in Fig. 2. Two patients were treated with PCM only, all other patients required stronger acting agents to relieve pain. In the TREO cohort, 11 out of 34 patients (32%) were treated with additional tramadol and 6 patients (18%) needed strong acting opiates (morphine, fentanyl). Strikingly, 15 patients (44%) needed adjuvant medicines (e.g. ketamine, clonidine, pregabalin or gabapentin, benzodiazepines) in order to manage pain adequately. Twelve patients (11%) experienced both myalgia and mucositis. In eight patients, mucositis was not severe (grade 2) and pain medication was intended for relieving myalgia only. In the other four patients, a strong acting opiate with one or more adjuvant medicines was started to relieve both mucositis and myalgia.

### Prognostic factors for development of myalgia

**Figure 3:**
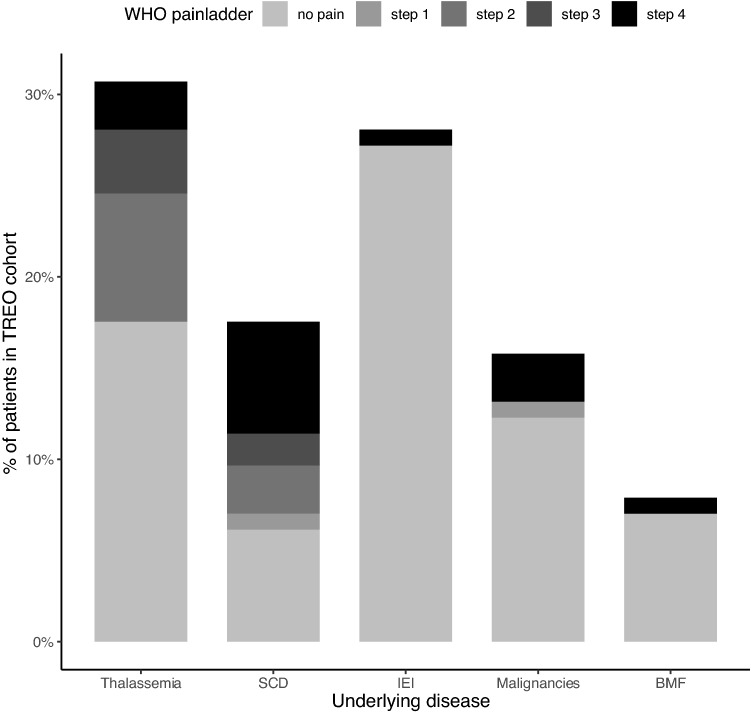
Pain severity (classified as steps in the WHO pain relief ladder) according to underlying disease in the TREO cohort (*SCD* sickle cell disease, *IEI* inborn errors of immunity, *BMF* bone marrow failure).

Prognostic factors were explored in the TREO cohort (Table 3). In univariable analysis, conditioning regimen, age, underlying disease and treosulfan exposure (AUC_0-∞_) were explored as possible factor. Multivariable logistic regression showed that underlying disease was a significant prognostic factor for the development of myalgia. Patients with hemoglobinopathy, especially patients with SCD, had a higher risk of experiencing myalgia than patients with other underlying diseases (OR 7.16 95%CI 2.09–30.03, p = 0.003). Thirteen of 20 patients (65%) with SCD experienced myalgia, half of them experiencing severe pain. It is important to note that the pain described by SCD patients was different from what they had previously experienced as disease specific pain (vaso-occlusive crises). Frequencies of pain severity per disease category are shown in Fig. 3. Furthermore, age proved a significant factor. Children above 3 years of age had a higher risk of experiencing myalgia than infants under 3 years old (OR 8.98 95% CI 2.04–64.54, p = 0.01). Conditioning regimen was not a significant covariate (OR 0.64 95% CI 0.14–2.92, p = 0.57 and OR 1.73 95% CI 0.05–71.17, p = 0.75 for treosulfan with fludarabine only or treosulfan with other agents, respectively). Treosulfan exposure (AUC_0-∞_) in bloodserum was available in 93 of 114 patients (82%). Treosulfan AUC_0-∞_ was not related with the occurrence of myalgia (p = 0.23).

**Table 3:** Univariable and multivariable analysis of the treosulfan cohort.

Variable	OR 95% CI	p-value	Adjusted OR 95% CI*	p-value
**Underlying disease**				
Hemoglobinopathy	9.16 (3.58–26.97)	< 0.001	7.16 (2.09–30.03)	0.003
**Age**				
> 3 years of age	10.3 (3.34–45.50)	< 0.001	8.98 (2.04–64.54)	0.01
**Conditioning regimen**				
Treo-Flu	0.28 (0.09–0.74)	0.02	0.64 (0.14–2.92)	0.57
Treo-Other	1.71 (0.07–44.50)	0.71	1.73 (0.05–71.17)	0.75
Treosulfan exposure (AUC_0-∞_) (for every 500 mg*hr/L increase in AUC_0-∞_)	0.34 (0.17–0.64)	0.002	0.61 (0.26–1.33)	0.23

*Treo* treosulfan, *Flu* fludarabine, *AUC* Area under the concentration curve.

Adjusted for underlying disease, age, conditioning regimen and treosulfan exposure.

## Discussion

This study provides an insight into the incidence, duration and severity of myalgia after treosulfan-based conditioning prior to pediatric HSCT. A substantial amount of the patients in the TREO-based cohort developed severe myalgia, especially in patients treated for hemoglobinopathy. This was not observed after BU-based conditioning. The comparison was made with a BU cohort, so that potential confounding of other factors, such as concomitant agents in the conditioning regimen (namely fludarabine, but also thiotepa), could be ruled out as much as possible.

To our knowledge, this is the first study addressing this serious side effect of treosulfan. In the original SmPC, myalgia was not mentioned as one of the side effects^13^. In the updated SmPC, pain in extremities is mentioned in the undesirable effects in the pediatric population with unknown frequency. For the adult population, myalgia and arthralgia are listed as common (1–10%) side effects. In our patients we found a higher incidence of 30%, predominantly in the hemoglobinopathy group.

A possible explanation could be the difference in the height of the dosages: for ovarian cancer, the dose is 5–8 g/m^2^ every 3–4 weeks, whereas for conditioning prior to HSCT a higher dose of 30–42 g/m^2^ is applied, divided over three consecutive days.

It is known that particular chemotherapeutic agents are associated with significant muscle and joint pains. These are agents that inhibit microtubular function (antimitotics, i.e. the vinca alkaloids, particularly vinblastine, vincristine and vindesine, and the taxanes, paclitaxel and docetaxel). The pathophysiologic mechanisms remain unclear, but it is thought that disruption of the microtubules, which are critical for maintenance of cell shape, motility and anchorage, mediation of signals between surface receptors and the nucleus and intracellular transport, cause cell death^17^. CK elevation is reported sometimes, while other case reports do not^18–21^. Treosulfan is not an antimitotic agent and its mechanism of action is different. In a study in rats done by Romanski et al., the disposition of treosulfan and its active monoepoxide in different organs (bone marrow, liver, lungs, brain and muscle) was investigated^22^. The study shows a comparable exposure of treosulfan in muscle and plasma, but a higher exposure of the active metabolite in muscle than in plasma. The authors mention that these findings may be explained by lower molecular weight (182 Da) and higher lipophilicity (logP − 1.18) of the metabolite. It is possible that this higher exposure ratio in muscle to plasma is responsible for damage in myocytes, causing myalgia. Busulfan can also cause myalgia, as reported in the adverse effects section of the SmPC^23^. However, in our study we found a significantly lower incidence in patients treated with busulfan-based conditioning compared to treosulfan-based conditioning. A possible explanation is that the standard daily doses of treosulfan that is applied in conditioning prior to HSCT (10–14 g/m^2^) are much higher than the doses used for busulfan (max. 3.2 mg/kg). The concentrations of treosulfan and its active monoepoxide are expected to be much higher than busulfan. In our study, we had data of treosulfan exposure in a majority of patients. A relationship between treosulfan exposure in serum and the occurrence of myalgia could not be found, in contrast to other early adverse events, such as mucositis and skin toxicity^24^. However, the AUC_0-∞_ in serum might not reflect the concentration of the metabolite in muscle, which could be different. The concentration of active monoepoxide could me more interesting, as this seems relatively high in muscle. CK elevation was not found. However, myalgia without CK elevation is not uncommon and is described in many drug-induced myopathies, such as statins^25^.

We found that underlying disease was a prognostic factor for the development of myalgia. Patients transplanted for beta-thalassemia and SCD are seven times more likely to develop myalgia than patients transplanted for other diseases. In the literature, no specific reports on myalgia are found. This could be due to lack of awareness of this (transient) adverse event as well as the low incidence in some categories of patients as shown in this study. The underlying cause of this correlation is currently unknown, but several hypotheses can be considered. Most SCD patients have a complex pre-transplant disease history, with systemic vasculopathy causing different complications involving pain. Their pain perception could be altered and this could be lead to myalgia being perceived as more painful than in other diseases. It is also possible that there is a genetic predisposition factor associated with the occurrence of myalgia after treosulfan administration. Wonkam et al. found pain-related genes correlated with vaso-occlusive crises (CACNA2D3-rs6777055, *P* = *0.025*; DRD2-rs4274224, *P* = *0.037*; and KCNS1-rs734784, *P* = *0.01*) in patients with sickle cell disease^26^. Thalassemia, and hemoglobinopathies in general, are more common in certain ethnic groups. It would be interesting to investigate if genes may be associated with this adverse effect. This could be addressed in future research.

There are some limitations to our study. We performed a retrospective database study and the validity of the data is dependent on the observations of the nursing staff and accuracy of reporting in the medical records. In the pediatric population, recognition and assessment of myalgia in babies and infants (< 3 years old) is difficult, because they are (mostly) unable to indicate the type of pain they are experiencing. This may have contributed to our observation that higher age appeared to be a significant prognostic factor. Also, it is possible that we underestimated the number of patients with myalgia and its severity, because the medical records were initially screened using the CDC. However, a recent study has shown that the use of this search engine is reliable and accurate^27^. Overestimation is highly unlikely, because all EHRs with a positive hit were screened and confirmed manually. Furthermore, due to the fact that myalgia was reported more frequently over time after treosulfan was introduced as a conditioning agent, it is possible that the nursing staff recognized and classified this type of pain better over time. This means that there is a possibility of underreporting in the first years after introduction of treosulfan. However, since the majority of patients required heavy pain medication to alleviate the pain, the risk of underreporting can be considered small. Another limitation is the imbalance in underlying diseases between the TREO and BU cohort. We found that hemoglobinopathies have a significantly higher risk to develop myalgia and this group is underrepresented in the BU cohort. As there were no SCD patients in the BU cohort, we cannot definitively rule out the possibility that myalgia would have occurred as well when SCD patients were conditioned with a BU-based regimen. However, the majority of published literature on transplantations of SCD patients are with BU-based regimens^28,29^. In those studies, myalgia has not been reported as an adverse event. Moreover, in our study there were no reports of myalgia in patients with beta-thalassemia in the BU cohort, whereas in the TREO cohort 15 out of 34 patients (44%) reported myalgia.

This study provides important new knowledge about treosulfan and its adverse events. The impact of myalgia on the patient’s experience during the SCT treatment can be significant. Patient and nursing staff education is an essential part of the nursing care plan to manage drug- or disease related arthralgias and myalgias. If patients and the nursing staff—in particular for babies and infants—recognize these symptoms and a plan is available to deal with the discomfort, therapeutic approaches can be initiated earlier and the quality of life of the transplanted patients is minimally affected. Furthermore, the use of an electronic health record text mining tool has proven to be helpful in tracking adverse events. More studies have been published using CTcue or a comparable tool to retrieve data from the EHR, including a validation study^27,30,31^. In the future, in case of a suspicion of the occurrence of a specific adverse event, a text mining tool can efficiently extract data from EHRs and can therefore quickly provide clarity on the relationship with the use of a particular drug.

## Conclusion

Myalgia is a common adverse effect in treosulfan-based regimens in pediatric patients in the setting of HSCT, particularly in hemoglobinopathies. This study shows that retrospective studies can make an important contribution to the knowledge and recognition of adverse events. It provides valuable information, that can be included in the Summary of Product Characteristics of treosulfan. A text mining tool such as the CDC can help to detect adverse events more efficiently. More research is needed to learn more about the mechanism of action and factors that influence the development of myalgia.

## Supplementary Information

Supplementary Information.

## Data Availability

The datasets generated during and/or analysed during the current study are available from the corresponding author on reasonable request.

## Code availability

The code for statistical analysis in R is available from the corresponding author on reasonable request.
