# Supplementary material for: Treosulfan-induced myalgia in pediatric hematopoietic stem cell transplantation identified by an electronic health record text mining tool
Source: Sci Rep. 2021 Sep 27;11:19084. doi: 10.1038/s41598-021-98669-3 (PMC8476488; doi:10.1038/s41598-021-98669-3)
Supplement: Supplementary file 1 — Supplementary Information. [file 41598_2021_98669_MOESM1_ESM.pdf]

Treosulfan-induced myalgia in pediatric hematopoietic stem cell transplantation identified by an electronic health record text mining tool.

M Y Eileen C van der Stoep[1,2], D Berghuis[2], Robbert GM Bredius[2], Emilie P Buddingh[2], Alexander B Mohseny[2], Frans J W Smiers[2], Henk-Jan Guchelaar[1], Arjan C Lankester[2], Juliette Zwaveling[1]

[1]Department of Clinical Pharmacy and Toxicology, Leiden University Medical Center, Leiden, The Netherlands, [2]Department of Pediatrics, Leiden University Medical Center, Leiden, The Netherlands

Correspondence

Eileen van der Stoep, Department of Clinical Pharmacy and Toxicology, Leiden University Medical Center, Leiden, PO Box 9600, 2300 RC Leiden, The Netherlands; telephone: +31 71 529 9282, fax: +31 71 529 6886, e-mail: e.vanderstoep@lumc.nl

Supplementary Table 1. Synonyms of myalgia used for screening EHRs

**Dutch**	
Myalgie	Spierpijn
Spier pijn	Pijn spier
Spierpijnen	Spier pijnlijk
Spieren pijn	Pijn spieren
Pijnlijke spier	Pijnlijk spieren
Spieren pijnlijk	Pijnlijke spieren
Gegeneraliseerde spierpijn	Gegeneraliseerd spierpijn
Spierpijnen gegeneraliseerd	Gegeneraliseerde spierpijnen
**English**	
Myalgia	Myodynia
Muscle pain	Muscle aches
Muscular pains	Muscle soreness
